# A Meta-Analysis of the Effects of Dietary Betaine on Milk Production, Growth Performance, and Carcass Traits of Ruminants

**DOI:** 10.3390/ani14121756

**Published:** 2024-06-11

**Authors:** Archana Abhijith, Frank R. Dunshea, Surinder S. Chauhan, Veerasamy Sejian, Kristy DiGiacomo

**Affiliations:** 1 School of Agriculture, Food and Ecosystem Sciences, The University of Melbourne, Parkville, VIC 3010, Australiafdunshea@unimelb.edu.au (F.R.D.); ss.chauhan@unimelb.edu.au (S.S.C.); 2 School of Biology, Faculty of Biological Sciences, The University of Leeds, Leeds LS2 9JT, UK; 3 Rajiv Gandhi Institute of Veterinary Education and Research (RIVER), Kurumbapet, Puducherry 605009, India; drsejian@gmail.com

**Keywords:** lactation, carcass, growth, nutritional strategy, sugar beet, betaine, diet supplementation

## Abstract

**Simple Summary:**

Betaine, a natural by-product of the sugar beet industry, can improve growth performance when fed to production animals, particularly under increased environmental temperatures. While the positive effects of betaine in monogastric species like pigs and poultry are well investigated, efficacy in ruminants is less clear. The increasing severity and frequency of heat events will impact livestock production, and dietary betaine offers a management tool to mitigate these events. This meta-analysis examined the effects of betaine supplementation on milk production, growth performance, and carcass traits of ruminants. The results showed a positive effect of dietary betaine supplementation in lactating dairy cattle, beef cattle, and sheep. These promising results show that dietary betaine supplementation can be utilized to improve productivity in ruminants under both ambient and hot conditions.

**Abstract:**

Betaine improves growth performance and health in monogastric animals under both thermoneutral and heat stress conditions, but results in ruminants have been more equivocal. This meta-analysis investigated the effects of betaine supplementation on productive performance, milk production and composition, and carcass traits of ruminants due to betaine supplementation. A comprehensive search for published studies investigating the effect of betaine was performed using Google Scholar, ScienceDirect, PubMed, and Scopus databases. Effect size analysis, random effects models, I^2^ statistics, and meta-regression analysis were utilized to assess differences in production parameters. Dietary betaine supplementation increased milk yield (+1.0 kg/d (weighted mean differences presented in this abstract), *p* < 0.001), dry matter intake (+0.15 kg/d, *p* < 0.001), and milk lactose (+0.05%, *p* = 0.010) in dairy cows housed under thermoneutral conditions. In the few studies conducted on small ruminants, there was an increase in milk yield in response to dietary betaine (0.45 kg/d, *p* = 0.040). Under heat stress conditions or grazing pasture during summer, dietary betaine increased milk yield (+1.0 kg/d, *p* < 0.001) and dry matter intake (+0.21 kg/d, *p* = 0.020). Dietary betaine increased final liveweight (+2.33 kg, *p* = 0.050) and back fat thickness (+0.74 cm, *p* < 0.001) in beef cattle. Dietary betaine increased final liveweight (0.14 kg, *p* = 0.010), daily gain (+0.019 kg/d, *p* < 0.001), and carcass weight (+0.80 kg, *p* < 0.001) but not backfat in small ruminants. These meta-analyses showed that dietary betaine increases liveweight in small ruminants and beef cattle and increases feed intake and milk yield in dairy cattle.

## 1. Introduction

The dietary supplement betaine is commonly fed to ruminants to act as an osmolyte, provide a source of methyl groups, and act as a molecular chaperone. These actions allow betaine to improve animal growth and productivity under both thermoneutral and heat stress conditions. As betaine can directly donate methyl groups, betaine supplementation can provide methyl groups and spare choline for use in other essential processes [1]. Betaine is a trimethyl derivative of the amino acid glycine, which is naturally produced as a by-product of sugar beet processing or produced endogenously by choline oxidation [2]. It is a popular feed additive for livestock, which is commercially available mostly as feed-grade in the form of anhydrous betaine, betaine monophosphate, and betaine hydrochloride [1]. Concentrated separator by-product (CSB) is also known to contain high concentrations of betaine [3], which also serves as a source of betaine for cattle diets. It is produced as a by-product of the sugar industry that results when additional sugar is extracted from beet molasses.

There are two main functions of betaine in the animal’s body. Firstly, it is an organic osmolyte that helps to reduce dehydration, stabilize protein structure, and preserve enzyme function when a cell is under osmotic stress. As a result, gastrointestinal integrity is enhanced [4] while maintenance energy expenditure is reduced [5]. Another important function of betaine is that it serves as a methyl donor and is a fundamental component in one-carbon metabolism [6]. When it donates a methyl group to the universal methyl donor S-adenosylmethionine, through methionine, betaine contributes to several critical functions in the body, such as growth, lactation, and liver health [7]. These actions have led betaine to be recommended as a dietary supplement to ameliorate heat stress (HS), particularly in monogastric animals [8], by reducing energy expenditure (and thus reducing metabolic heat loads), protecting gut tissues, and maintaining osmotic balance [1].

Various researchers have found that up to 150 g/day of dietary betaine increases milk yield in a linear manner under thermoneutral conditions [9,10,11]. However, the effects of dietary betaine on lactating dairy cows during summer or under hot conditions are more equivocal [9,12], perhaps because of the multifaceted dose-dependent responses to betaine [13]. For example, anhydrous betaine supplementation reduced fat thickness in female but not male sheep [14], while in dairy cattle betaine supplementation improved milk yield in dairy cattle when fed during thermoneutral but not heat stress conditions [9]. Similarly, responses in non-lactating ruminants and monogastric animals have been variable. To unravel some of the variability in response in pigs, Sales et al. [15] conducted a meta-analysis of published data, which helped to demonstrate the overall efficacy of dietary betaine in pigs. While widely used in the animal nutrition literature, qualitative reviews are mostly subjective in nature and therefore inclined to reviewer’s bias [16]. Conclusions are determined based on the results of null hypothesis significance testing without considering the measures of dispersion among studies [17]. The meta-analysis technique offers an alternative approach that can take account of this variation and provide quantitative estimates of the impact of an intervention. Therefore, the current meta-analysis aimed to investigate the effects of betaine supplementation on milk production, growth performance, and carcass traits of ruminants using meta-analytic techniques.

## 2. Materials and Methods

### 2.1. Literature Search

**Figure 1:**
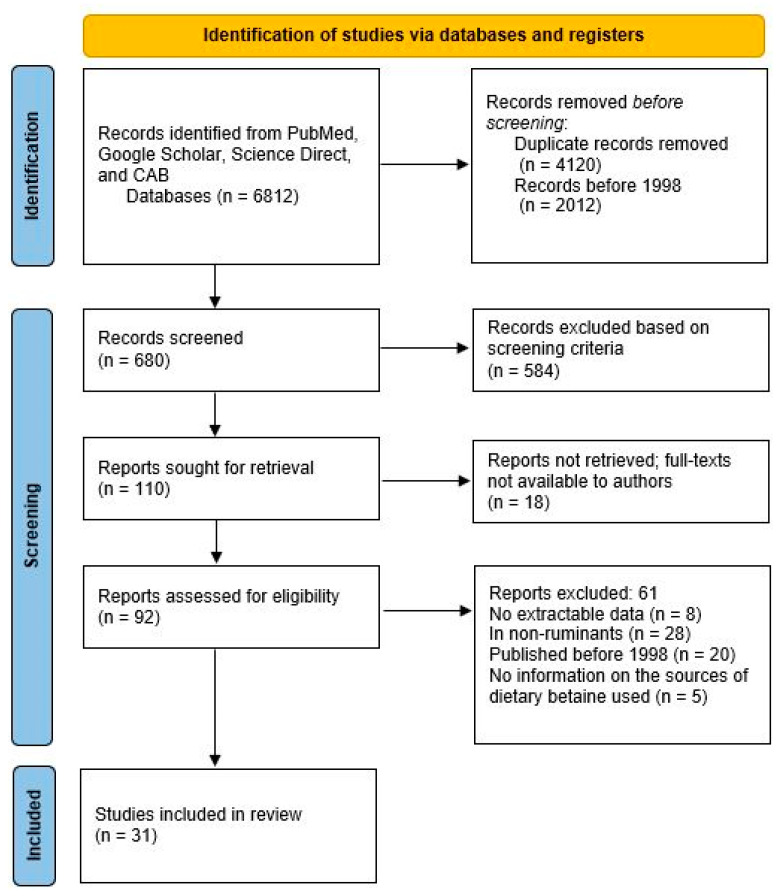
PRISMA flow diagram for selection of records included in the meta-analysis. Source: [18].

The research questions, search and screening protocols, and result reporting followed the Preferred Reporting Items for Systematic Reviews and Meta-Analyses (PRISMA) guidelines [18]. A comprehensive search for published studies (Figure 1) on betaine effects on growth, milk production, and carcass characteristics was performed using PubMed, Google Scholar, Science Direct, and CAB, and identification of other studies from references lists in papers. Searches were based on the following keywords: betaine, CSB, betafin, sugarcane molasses, feed supplement, cattle, and beef, using the terms for the product brand names separately with the term ruminant, goat, sheep, cattle, and beef.

### 2.2. Paper Inclusion/Exclusion Criteria

The following inclusion criteria were used to build a database of research papers: full-text manuscripts from peer-reviewed journals; betaine dose and source were clearly stated; period of experiment, animals studied were ruminants; the paper contained sufficient data to determine the effect size for production outcomes (e.g., the number of cattle, or carcasses in each treatment and control group); a measure of effect amendable to effect size analysis for continuous data (e.g., standardized mean difference, SMD); a measure of variance (SE or SD) for each group. Papers were excluded from the analysis if they did not meet all the required criteria. The inability to separate results for different treatments and to obtain the required information from the authors excluded some studies. The mean values of control and treatment groups, the number of samples allocated to control and treatment, and a measure of variance expressed either as standard error (SE) or standard deviation (SD) were kept as the required data that were noted from all the selected papers for the meta-analysis. The papers comprising the database are summarized in Supplementary Table S1. We also used an unpublished data set from our own laboratory, which satisfied all criteria except it had not been peer-reviewed. The use of such grey data has been justified for use in meta-analysis as it can often reduce publication bias since unpublished data often have smaller effect sizes, and there is no difference in the robustness of the studies [19].

### 2.3. Data Set

The database comprises author names with year, where possible, breed, age and gender, dose, and period of betaine supplementation. The data were exclusively on the growth, milk, and carcass characteristics of ruminants. A data set of 31 studies with 56 experiments between 1998 and 2021 was obtained that fulfilled the required criteria. Some studies have not reported information on the sources of dietary betaine used, which eliminated any standardization for purity. Anhydrous betaine was used in most studies, including [14,20,21]: 98% purity, [22,23]: 96% purity, [9]: 93%, [10,12],: 97% purity, [24]: 30% purity. Other forms included betaine hydrochloride [25,26], and natural betaine [27,28]. Monteiro et al. [29] used liquid supplement made of molasses from sugar cane and condensed beet solubles as a betaine source.

The diets included milo-soyabean [20], wheat [22], corn [3,12,14,24,30], maize-soyabean [21,31,32,33], alfalfa [9,10], and barley [14].

Outcomes were reported inconsistently among individual experiments. Final body weight (FBW), dry matter intake (DMI), average daily gain (ADG), 12th rib fat thickness (referred to as backfat thickness), milk yield, milk fat %, milk fat yield, milk lactose %, milk lactose yield, milk protein %, milk protein yield, and hot carcass weight (HCW) were the only outcomes that could be considered for analyses. The average period that animals were fed betaine was 71 days.

### 2.4. Data Analysis

The meta-analysis was conducted using Comprehensive Meta-analysis software (CMA) Version 3, Biostat, Englewood, NJ, USA, [34] to analyze growth, milk production, and carcass data by SMD which is also called effect size (ES) analysis, in which the difference between treatment and control group means was standardized using the standard deviations of control and treatment groups. Responses are presented as weighted mean differences unless otherwise stated.

Random effects models were used to estimate the effect size, 95% confidence intervals, and statistical significance of SMD for each outcome. It should be emphasized that SMD are dimensionless measures of effect, independent of differences in unit measurements and sample sizes [35]. Raw and weighted mean differences are also shown to provide differences in dimensions. Estimated effect sizes were visually presented using forest plots, with data selected for this method of presentation based on significance (*p* value). The upper and lower limit of the line connected to the square shows the upper and lower 95% confidence interval (CI) for the effect size. The vertical line in the middle of the plot represents the mean difference from zero or the line of no effect. Points to the left of the vertical line represent a reduction in the outcome, whereas points to the right of the line indicate an increase in the outcome variable. Effect sizes were categorized according to Cohen et al. [36], as small, medium, and large at values of 0.2, 0.5, and 0.8, respectively.

Asymmetry of the funnel plots was used to study publication biases [37]. Funnel plots are simple scatter plots of the treatment effect estimates from individual studies plotted against study precision. Effect estimates from small studies will scatter widely at the bottom of the plot, and the spread will be narrow for larger studies. The plot resembles a symmetrical (inverted) funnel in the absence of bias. Smaller studies without statistically significant effects usually remain unpublished; this will lead to an asymmetrical appearance of the funnel plot. A gap will be evident towards the bottom of the funnel plot in such cases. The effect calculated in such a situation tends to overestimate the intervention effect.

Heterogeneity between the studies indicates differences in study design, differences in clinical diversity of the herds, level/source of betaine used, and analytical methods and statistical variation around responses. We used I^2^ statistic to assess heterogeneity; I^2^ < 30% was considered to be mild heterogeneity, 30–50% as moderate, and >50% as severe heterogeneity [38]. This means that only I^2^ was the true heterogeneity, and the remaining was due to the sampling errors of individual studies. We used meta-regression analyses to explore the source of heterogeneity of response, using the SMD for each trial as the outcome and the associated standard error as the measure of variance. This helps to explore reasons for heterogeneity and as a function of the a priori defined covariate changing. The inclusion of multiple covariates in the meta-regressions was used [39].

## 3. Results

Mean effect sizes (SMD) calculated according to a random effects model for the different outcome variables are shown in Table 1, Table 2 and Table 3. Based on visual assessments from the output provided by the statistical analysis, the ideal dose of betaine appears to be approximately 0.125 g/LWT0.75.

### 3.1. Milk Yield

Under thermoneutral or prevailing ambient conditions, dietary betaine increased milk yield (+1.0 kg/d, *p* < 0.001), dry matter intake (DMI, +0.15 kg/d, *p* < 0.001), and milk lactose (+0.05%, *p* = 0.010) and tended to increase milk fat yield (+7.0 g/d, *p* = 0.070) in dairy cows (Table 1). There were no other significant effects on either the % or yields of other milk constituents, perhaps because of fewer comparisons, at least for the yields. In the small number of studies conducted on small ruminants, there was an increase in milk yield in response to dietary betaine (0.45 kg/d, *p* = 0.040).

**Figure 2:**
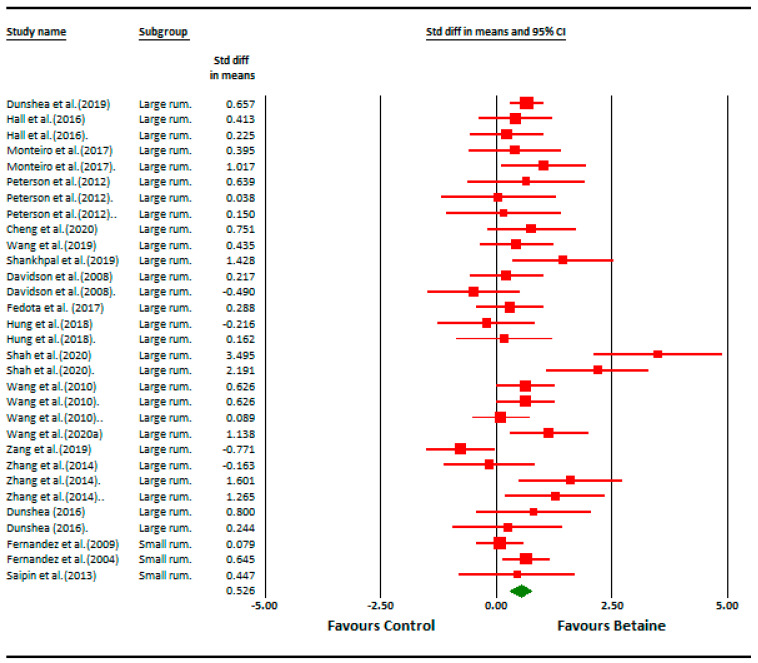
Forest plot of milk yield responses for betaine studies. A Forest plot of the effect size or standardized mean difference (standardized using the z-statistic) and 95% confidence interval of the effect of betaine treatment milk yield. The larger the box, the greater the study’s contribution to the overall estimate. The solid vertical black line represents a mean difference of zero or no effect. Points to the left of the line represent a reduction in milk yield, while points to the right of the line indicate an increase. The upper and lower limit of the line connected to the square represents the upper and lower 95% confidence interval for the effect size. The overall pooled effects size and 95% confidence interval is indicated by the diamond at the bottom. This effect was very slightly heterogeneous as indicated by the I^2^ of 10% for large ruminants and 15% for small ruminants (Table 1) [9,10,11,12,21,26,28,29,30,31,33,40,41,42,43,44,45,46].

**Figure 3:**
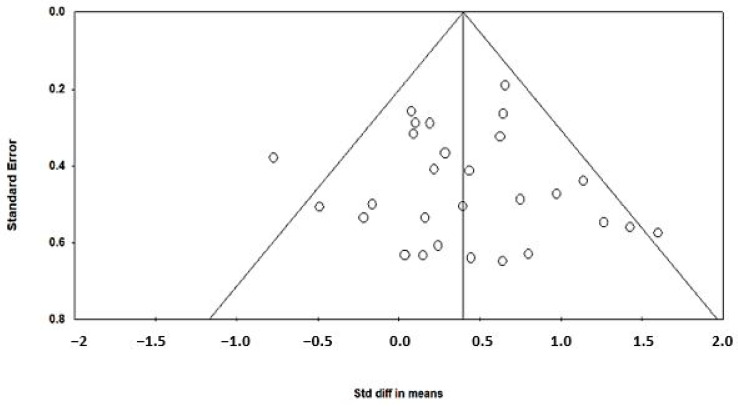
Funnel plot of standard error against standardized difference in means of effects of dietary betaine on milk yield in dairy cattle and small ruminants under either thermoneutral or ambient conditions.

**Figure 4:**
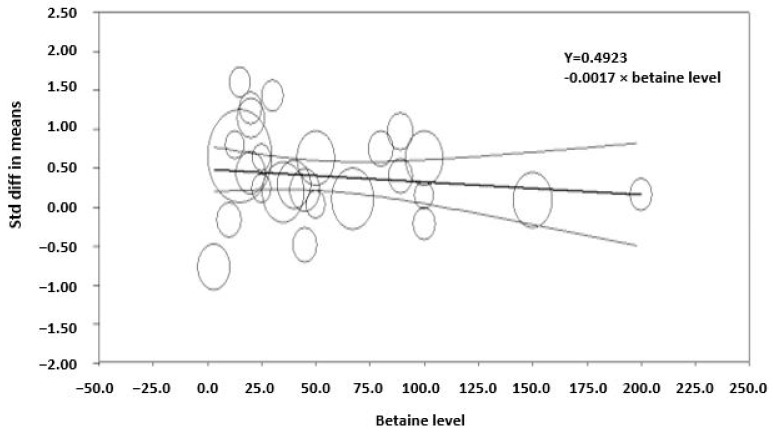
Meta-regression of the effect of the level of betaine supplementation on the standardized mean difference in studies examining betaine and milk yield in lactating animals (*R*^2^ = 33, *p* < 0.001). The regression is weighted by the effect size of studies, which is indicated by the size of the marker. The larger the marker, the greater the effect size of the study.

**Figure 5:**
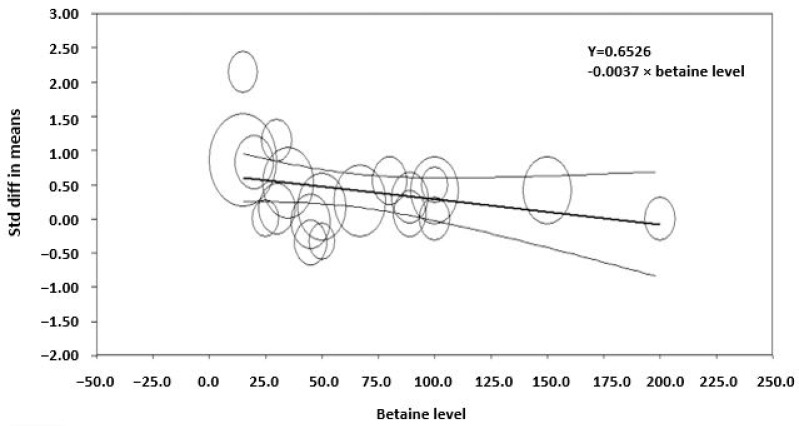
Meta-regression of the effect of the level of betaine supplementation on the standardized mean difference in studies examining betaine and dry matter intake in lactating animals (*R*^2^ = 74, *p* < 0.001). The regression is weighted by the effect size of studies, which are indicated by the size of the marker. The larger the marker, the greater the effect size of the study.

The effect of dietary betaine on the SMD of milk yield for individual studies pooled across large and small ruminants indicates an effect size of 0.53, which is considered a medium effect (Figure 2). Importantly, a symmetrical scatter of points on both sides of the standardized mean difference (symmetrical funnel plot) was observed for milk yield, indicating a probable absence of publication bias (Figure 3). The effect of dietary betaine on milk yield (Figure 4) and DMI (Figure 5) decreased with increasing doses of dietary betaine, as indicated by the significant meta-regressions.

**Figure 6:**
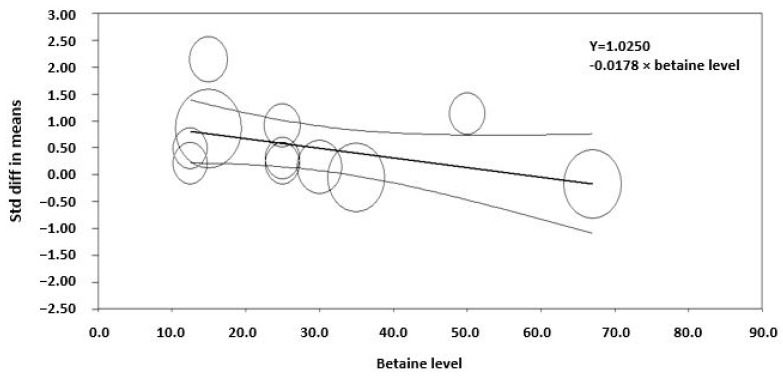
Meta-regression of the effect of the level of betaine supplementation on the standardized mean difference in studies examining betaine and dry matter intake in lactating animals during heat stress (*R*^2^ = 0.84, *p* < 0.001). The regression is weighted by the effect size of studies, which are indicated by the size of the marker. The larger the marker, the greater the effect size of the study.

**Figure 7:**
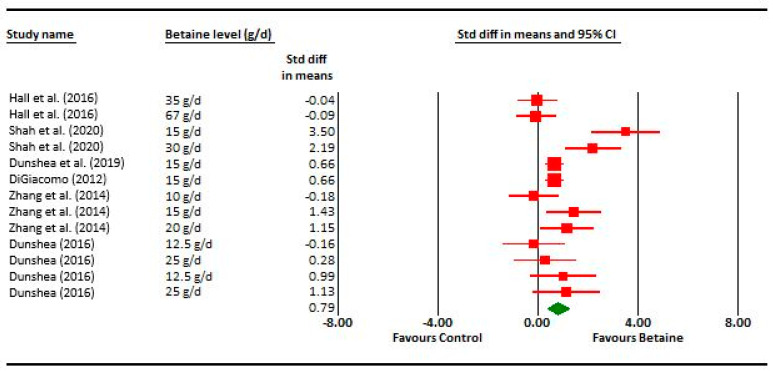
Forest plot of milk yield responses for betaine studies during heat stress. A Forest plot of the effect size or standardized mean difference (standardized using the z-statistic) and 95% confidence interval of the effect of betaine treatment on milk yield during heat stress. The larger the box, the greater the study’s contribution to the overall estimate. The solid vertical black line represents a mean difference of zero or no effect. Points to the left of the line represent a reduction in milk yield, while points to the right of the line indicate an increase. The upper and lower limit of the line connected to the square represents the upper and lower 95% confidence interval for the effect size. The overall pooled effects size and 95% confidence interval is indicated by the diamond at the bottom [9,12,27,28,30,42].

Dietary betaine increased milk yield (+1.0 kg/d, *p* < 0.001), DMI (Figure 6; +0.21 kg/d, *p* = 0.020), and tended to reduce milk fat % (−0.13%, *p* = 0.060) in dairy cows housed under heat stress conditions or grazing pasture during summer (Table 2). There were no other significant effects on milk components. The effect of dietary betaine on the SMD of milk yield for individual studies pooled indicates an effect size of 0.79, which is considered a medium to large effect (Figure 7).

### 3.2. Beef Cattle

Dietary betaine tended to increase DMI (+0.15 kg/d, *p* = 0.080) and consequently increased final liveweight (+2.33 kg, *p* = 0.050) and back fat thickness (+0.74 cm, *p* < 0.001) in beef cattle (Table 3). Dietary betaine increased final liveweight (+0.14 kg, *p* = 0.010), daily gain (+0.019 kg/d, *p* = <0.001), and carcass weight (+0.80 kg, *p* < 0.001) but not backfat in small ruminants (Table 3). There were insufficient studies to determine the effect of dietary betaine on the growth performance of beef cattle or small ruminants under HS conditions.

## 4. Discussion

This quantitative meta-analysis of data from several experiments revealed that betaine supplementation significantly improved milk yield and dry matter intake in lactating ruminants under both thermoneutral and heat stress conditions. The effect size of betaine treatment on milk yield, DMI, and milk lactose % was substantial, with an increase of approximately 1 kg/d, 0.15 kg/d, and 0.05%, respectively, in the supplemented group compared to their controls. The very low heterogeneity in milk yield and DMI responses indicates that this is a consistent response, providing statistical proof of increased milk yield and DMI in dairy ruminants supplemented with betaine.

Dietary betaine also improved growth rate and carcass weight in growing ruminants, although the effect sizes were smaller. It appears that the increase in daily gain and carcass weight was in part driven by an increase in backfat thickness, i.e., fat (rather than protein) accretion. Under conditions where energy intake is limiting protein deposition, such as rapidly growing pigs or poultry, any energy spared via the reduction in ion pumping due to the osmoprotectant role of betaine can be used for protein and muscle deposition [47,48]. However, in animals consuming energy more than that required to maximize protein deposition, the energy is partitioned towards fat deposition [48]. This is most likely the reason for the increase in backfat thickness in cattle supplemented with betaine.

The ability of betaine to reduce heat stress has been reported previously in pigs [49] and chickens [4,47,50,51]. However, the effects of betaine on milk yield during summer or heat stress conditions are more equivocal. The mechanism of this difference in responses is yet to be elucidated, although some potential mechanisms have been hypothesized. Xiao et al. [52] conducted a detailed study of bovine mammary cells exposed to thermal shock and concluded that betaine serves as a chemical chaperone to restore secondary structures of mitochondrial enzymes (higher mitochondrial dehydrogenase activity) and helps to maintain the functionality in mammary epithelial cells during heat stress (higher HSP70 and HSP27 expression).

There are several studies that have shown that thermal stress impacts the somatotrophic axis of the animal and decreases circulating somatropin [53,54]. Conversely, the positive influence of betaine on increasing plasma somatotropin concentrations [55,56] has been well documented. Also, Dangi et al. [57] reported that betaine supplementation reduces heat shock protein concentrations in goats. The author’s observation was that the amelioration effect of betaine in reducing the impact of heat stress, by increasing the growth hormone levels and other growth performance traits.

There was a variable degree of heterogeneity in response of some parameters to betaine between studies, so the sources of variation were explored using a meta-regression approach. The meta-regression analyses indicated that the response decreased with increasing dose with the milk yield and feed intake responses appearing to be optimized at the lower end of the doses investigated (between 10 and 15 g/d) for dairy cattle. This was evident in a study by Zhang et al. [12], where the researchers showed that milk yield increased at a dose of 15 g/d during summer, beyond which no effect was observed. These findings are consistent with the findings of DiGiacomo et al. [58,59] and DiGiacomo [27] who found that dietary betaine at 2 g/d (0.125 g/LWT0.75) was protective against heat stress in growing sheep. The moderation in response to high doses of betaine during heat stress was speculated to be due to the stimulation of hepatic metabolism and a consequent increase in heat production by the liver, which may offset the decrease in heat production by betaine in the rest of the body [28].

## 5. Conclusions

In conclusion, these meta-analyses show that dietary betaine increases feed intake, milk yield, and carcass weight in ruminants in a dose-dependent manner. Milk composition is little changed by dietary betaine. The responses to betaine supplementation appear to decrease with increasing doses.

## Figures and Tables

**Table 1:** Effect of dietary betaine on lactation performance in dairy cattle and small ruminants under either thermoneutral or ambient conditions.

	*n*	SMD (95% CI)	Raw Mean Difference	Weighted Mean Difference	I^2^ (%)	*p*-Value
Dairy cattle						
Milk yield, kg/d	27	0.51 (0.26, 0.76)	1.00	1.00	10	<0.001
Dry matter intake, kg/d	20	0.44 (0.24, 0.64)	0.20	0.15	21	<0.001
Lactose, %	22	0.40 (0.08, 0.63)	0.05	0.05	55	0.010
Lactose, g/d	11	0.13 (−0.17, 0.43)	7.50	5.94	25	0.39
Protein, %	23	0.05 (−0.25, 0.34)	0.00	0.01	41	0.75
Protein, g/d	14	0.24 (−0.18, 0.66)	6.40	6.40	0	0.26
Fat, %	26	−0.04 (−0.42, 0.34)	0.09	0.12	62	0.80
Fat, kg/d	15	0.32 (−0.03, 0.66)	8.50	7.00	59	0.070
Small ruminants						
Milk yield, kg/d	3	0.40 (0.02, 0.71)	0.50	0.45	15	0.040

**Table 2:** Effect of dietary betaine on lactation performance in dairy cattle under either heat stress or summer conditions.

	*n*	SMD (95% CI)	Raw Mean Difference	Weighted Mean Difference	I^2^ (%)	*p*-Value
Dairy cattle						
Milk yield, kg/d	13	0.96 (0.46, 1.47)	1.00	1.00	60	<0.001
Dry matter intake, kg/d	10	0.50 (0.05, 0.90)	0.30	0.21	60	0.020
Protein, %	14	0.20 (−0.12, 0.49)	0.001	0.001	44	0.23
Protein, g/d	6	0.10 (−0.15, 0.32)	16.0	12.0	0	0.47
Fat, %	14	−0.50 (−1.01, 0.34)	−0.14	−0.13	70	0.060
Fat, kg/d	6	−0.06 (−0.30, 0.17)	−9.0	−13.6	0	0.60

**Table 3:** Effect of dietary betaine on growth performance of beef dairy cattle and small ruminants under either thermoneutral or ambient conditions.

	*n*	SMD (95% CI)	Raw Mean Difference	Weighted Mean Difference	I^2^ (%)	*p*-Value
Beef cattle						
Dry matter intake, kg/d	11	0.23 (0.03, 0.47)	0.20	0.15	54	0.080
Final liveweight, kg	18	0.20 (0.01, 0.43)	4.40	2.33	70	0.050
Daily gain, kg/d	18	0.10 (−0.14, 0.28)	0.05	0.05	69	0.27
Carcass weight, kg	8	0.13 (−0.06, 0.32)	1.71	2.58	44	0.17
Fat thickness, mm	13	0.84 (0.30, 1.39)	0.73	0.74	57	<0.001
Small ruminants						
Final liveweight, kg	7	0.10 (0.04, 0.20)	0.13	0.14	50	0.010
Daily gain, kg/d	6	0.86 (0.78, 0.98)	0.019	0.019	70	<0.001
Carcass weight, kg	4	0.12 (−0.17, 0.43)	0.80	0.80	54	<0.001
Fat thickness, mm	8	0.25 (−0.5, 1.01)	0.10	0.10	23	0.54

## Data Availability

All relevant data are provided within the article.

## Supplementary Materials

The following supporting information can be downloaded at https://www.mdpi.com/article/10.3390/ani14121756/s1. Table S1: Summary of references and experimental design for the experiments included in the meta-analysis. References [3,9,10,11,12,14,20,21,22,23,24,25,26,28,29,30,31,33,35,37,40,41,43,44,45,46,58] are cited in the supplementary materials.
