# Supplementary material for: A Meta-Analysis of the Effects of Dietary Betaine on Milk Production, Growth Performance, and Carcass Traits of Ruminants
Source: Animals (Basel). 2024 Jun 11;14(12):1756. doi: 10.3390/ani14121756 (PMC11201161; doi:10.3390/ani14121756)
Supplement: Supplementary file 1 [file animals-14-01756-s001.zip › animals-2936081-supplementary.pdf]

Supplementary Table S1. Summary of references and experimental design for the experiments included in the meta-analysis.

Reference	Species and number	Dose(s) fed	Study design
Bock et al (2004)	Steers, n=80	20 g/h/d	2 × 2 factorial
	Steers, n=565	40 g/h/d	2 × 1 factorial
Cheng et al (2020)	Dairy cows, n=36	4 g/kg DM	Randomized block, 2 × 2 factorial
Davidson et al (2008)	Dairy cows, n=80	45 g/h/d	4 × 1 factorial
Dong et al (2020)	Lambs, n=60	1.1 g/d unprotetcted; 1.1, 2.2 or 3.3 g/d protected	5 x 1 factorial
Dunshea et al (2019)	Dairy cows, n=118	2 g/kg DM	2 × 1 factorial
DiGiacomo et al (2016)	Sheep, n=36	2 or 4g/h/d	3 × 2 factorial
DiGiacomo et al (2014)	Steers, n=48	10, 20 or 40 g/h/d	4 × 2 factorial
Fedota et al (2017)	Dairy cows, n=35	40 g/h/d	2 × 1 factorial
Fernández et al. (2000)	Lambs, n=60	2 g/kg DM	3 × 2 factorial
Fernández et al. (2004)	Dairy goats, n=30	4 g/kg DM	2 × 1 factorial
Fernández et al. (2009)	Dairy goats, n=60	4 g/kg DM	2 × 1 factorial
Gralak et al (1998)	Calves, n=30	2.5% DM	3 × 1 factorial
Hall et al (2016)	Dairy cows, n=24	57 or 114 mg/kg BW	3 × 1 factorial
Hung (2018)	Dairy cows, n=20	100 or 200 g/h/d	3 × 3 Latin square
Lakhani et al (2020)	Heifers, n=18	25 or 50 g/h/d	3 × 1 factorial
Liu et al (2021)	Bulls, n=44	0 or 0.6 g/kg DM	2 × 2 factorial
Loest et al (1998)	Steers, n=175	10.5 or 21 g/h/d; 15.5 or 31 g/h/d CSB betaine	Randomized block, 5 × 1 factorial
Loest et al (2001)	Heifers, n=300	4.2 g/h/d or 15.5 g/h/d CSB betaine	Randomized block, 3 × 3 factorial
Loest et al (2002)	Steers, n=175	10.5 or 21 g/h/d betaine; 15.5 or 31 g/h/d CSB betaine	Randomized block, 4 × 1 factorial
	Heifers, n=312	4, 8 or 12 g/h/d	Randomized block, 2 × 4 factorial
Monteiro et al (2017)	Dairy cows, n=20 and n=16	89.1 g/kg DM	2 × 1 factorial
Nezamidoust et al (2014)	Lactating ewes, n=20	5 g/kg DM	3 × 1 factorial
Peterson et al (2012)	Dairy cows, n=18	25, 50 or 100 g/h/d	4 × 4 Latin square
Saipin et al (2013)	Dairy goats, n=10	4 g/kg DM	2 × 1 factorial
Shah et al (2020)	Dairy cows, n=30	15 or 30 g/h/d	3 × 1 factorial
Shankhpal et al (2019)	Lactating buffaloes, n=24	30 g/h/d	3 × 1 factorial
Wang et al (2010)	Dairy cows, n=20	50, 100 or 150 g/h/d	4 × 4 Latin square

Wang et al (2019)	Dairy cows, n=24	20 g/h/d protected	2 × 1 factorial
Wang et al (2020a)	Dairy cows, n=36	20 g/h/d protected	3 × 1 factorial
Wang et al (2020b)	Bulls, n=48	0.6 g/kg DM	2 × 2 factorial
Zang et al (2019)	Dairy cows, n=30	3 g/h/d protected	2 × 1 factorial
Zhang et al (2014)	Dairy cows, n=32	10, 15 or 20 g/h/d	4 × 1 factorial

*note number of animals (n) includes all animals in the experiment and not total per betaine treatment. CSB = concentrated separator byproduct. Protected = rumen protected betaine.
